# San Antonio’s New Hospital

**Published:** 1901-06

**Authors:** 


### TEXAS MEDICAL JOURNAL.

AUSTIN, TEXAS.

A MONTHLY JOURNAL OF MEDICINE AND SURGERY.

EDITED AND PUBLISHED BY
F. E. DANIEL, M. D.

ASSOCIATE EDITOR:
WITTEN BOOTH RUSS, M. D.,
San Antonio, Texas.

Published Monthly at Austin, Texas. Subscription price $1.00 a year in advance.

Eastern Representative: John Guy Monihan, St. Paul Building, 220 Broadway, New York City.

Official organ of the State Association of Health Officers, the West Texas Medical Association, the Houston District Medical Association, the Austin District Medical Society, the Brazos Valley Medical Association, the Galveston County Medical Society, and several others.

### SAN ANTONIO'S NEW HOSPITAL.

Recently, by the efforts of two local specialists, a hospital and free clinic for the treatment of diseases of the eye, ear, nose and throat has been opened in San Antonio. Several prominent general practitioners are named as consulting physicians and surgeons, and a rather large number of ladies have been enlisted in the work of advertising the institution and of raising money for its support.

The work of organization has been done along strictly ethical lines and we have reason to know that the promotors are gentlemen of the highest professional attainments, and in good standing with the profession at large. These facts, however, do not make it any the less evident that no good excuse exists for establishing such an institution in a town the size of San Antonio, and it should require very little argument to prove that the establishment of free clinics, or of even partially free clinics, where they are not needed is sure to react most unfavorably upon the profession in the localities where they operate. In the large cities, where most of the hospitals offer free service to all comers, practically every branch of medicine and surgery is suffering materially from hospital competition. It is estimated that in New York, Philadelphia and other of the larger cities there are treated *free* more than five patients amply able to pay a physician to every one really deserving poor patient.

San Antonio, like most other towns of its size, is amply well provided with hospital facilities for the care of its destitute poor, and there is scarcely a general practitioner or specialist in the city who is not glad to assist in their treatment, *gratis,* at the charity institution provided by the city and county. Therefore, it seems evident that no free clinics run by private parties are needed, and that all such are a distinct menace to the welfare of the profession.

---

Dr. H. A. West, Secretary, is the representative of the Texas State Medical Association on the general committee of the American Medical Association to devise a plan of uniform organization. The committee will report at the St. Paul meeting, and doubtless their report will be adopted. Dr. West believes that "when the contemplated changes go into effect the evils under which we have labored heretofore will, to a great extent, be remedied, and the State Association will at once enormously increase in membership." So mote it be.

---

An "Interesting Subject."—During the discussion on "Malarial Hematuria," at Calvert meeting Brazos Valley Medical Society (see report herewith), on a warm afternoon—just after dinner, Dr. Thompson, of "McBurney's Point," was asleep by an open window through which came a refreshing breeze (he says he was *not* asleep; but that he hears better with his mouth open), was suddenly called on to *ante.* He started,—got up slowly, and, rubbing his eyes, said: "Mr. President, this is a very interesting subject..' And somehow they all laughed. Thompson told me afterwards that he was dreaming of fishing, and that while he was momentarily expecting a bite, there was a confusion of sounds—a buzzing—and he would catch, now and then, "hematuria," "hemoglobinuria," "quinine," "blood in the urine," "microscope"—here he got a bite, and jerked, just as they called on him to discuss; hence, he said, the start.

---

The Hot Stuff.—The "Red-Back's" State Agent, Mrs. S. Terry, a handsome young widow,—a blonde,—attends all the meetings of the medical societies in Texas. At the recent meeting of the Brazos Valley Medical Society, at Calvert, her table at the entrance of the hall was covered, as usual, with the current number of the Journal, and also with the *New Medical Practice Law in Texas,* "pamphlet form with remarks" red cover; and with *"Recollections of a Rebel Surgeon,"* in red. The "boys" called it the "chili stand," and the Secretary said: "Boys, step up and get some of the hot stuff." They all stepped up but two; one was lame in the foot and couldn't step, and the other one was broke.

---

The Commencement Exercises of the Medical Department of Tulane University were held at the Grand Opera House in New Orleans, May 1, 1901.

There were 126 graduates in medicine and eleven graduates in pharmacy. The exercises were notable for the fact that the usual valedictory was omitted and form the extraordinary features of the occasion in the introduction of the new president into the graduating ceremonies and in the marked distinction conferred upon the Dean, Prof. Stanford E. Chaillé, upon whom was conferred the degree of LL. D. by Tulane University. The friends of the Dean, and the *Journal* among them, are all free to congratulate Prof. Chaillé upon this recognition of his long service to the institution he has in so large degree fostered. His connection with the Medical Department since 1858 as a teacher, professor and dean was duly noticed in becoming resolutions from the faculty and from the student body. The recipient of the several tokens of esteem delivered himself of a characteristic speech of acceptance of these.

The address was delivered by the Hon. J. Hannis Taylor, on "The Relation of the Medical Profession to International Law."—*N. O. M. and S. Journal.*

